# The association of migration experiences on the self-rated health status among adult humanitarian refugees to Australia: an analysis of a longitudinal cohort study

**DOI:** 10.1186/s12939-019-1033-z

**Published:** 2019-08-22

**Authors:** Alison Dowling, Joanne Enticott, Marina Kunin, Grant Russell

**Affiliations:** 1 Department of General Practice, School of Primary Health Care, Monash University, Melbourne, Australia; 2 Southern Synergy, School of Clinical Sciences at Monash Health, Monash University, Melbourne, Australia

**Keywords:** Refugees, Humanitarian, Self-rated health, General health, Migration, Resettlement, Longitudinal, Building A new life in Australia

## Abstract

**Background:**

Refugees are potentially at an increased risk for health problems due to their past and current migration experiences. How migration factors shape refugee health is not well understood. We examined the association between migration factors and the self-rated general health of adult humanitarian refugees living in Australia.

**Methods:**

We analyzed the first three waves of data from the ‘Building A New Life In Australia’ longitudinal survey of 2399 humanitarian refugees resettled in Australia. The study outcome was self-rated health measured by the 36-Item Short Form Health Survey. Predictors were migration process and resettlement factors. We used generalized linear mixed models to investigate the relationship between predictor and outcome variables.

**Results:**

Poor general health persisted among this refugee population at high levels throughout the three-year follow-up. At baseline, 35.7% (95% CI: 33.8–37.7%) of the study population reported poorer general health. Female gender, increasing age and post-migration financial stressors were positively associated with poorer general health. Having a university degree and absence of chronic health conditions were seemingly protective against declining general health (OR: 0.50; 95% CI: 0.65–1.81 and OR: 0.15, 95% CI: 0.09–1.04, respectively).

**Conclusion:**

Our results show that there is persisting high prevalence of poorer general health among adult refugees across the initial years of resettlement in Australia. This finding suggests unmet health needs which may be compounded by the challenges of resettlement in a new society, highlighting the need for increased clinical awareness of this sustained health burden to help inform and prepare refugee health care and settlement service providers.

**Electronic supplementary material:**

The online version of this article (10.1186/s12939-019-1033-z) contains supplementary material, which is available to authorized users.

## Background

In 2017, almost 68.5 million people have been forcibly displaced from their homes, the largest number ever recorded. Of these, 25.4 million were recognised as refugees, while 3.1 million were asylum seekers [1]. Refugees are often unable to return to their home country for fear of death or persecution and may be offered resettlement in a third country [1], such as Australia, which offers resettlement to 18,750 refugees and others with humanitarian needs each year [2]. Refugees who are accepted for resettlement are among the most vulnerable groups in our society in terms of risk for poor health due to their past and current experiences [3]. Therefore, it is important for resettlement nations to understand the long-term health needs and settlement prospects of this vulnerable and ever-growing population, so that timely and appropriate supports can be provided [4–9]. However, the extent to which migration factors shape the long-term health of resettling refugees is not well understood.

Some of this knowledge gap can be attributed to methodological limitations in the current research [3]. To date, the bulk of the research investigating the health of resettling refugees has largely employed a cross-sectional methodology to investigate mental health outcomes, using a variety of quantitative measurement tools [3]. This has meant that information is not available on the full spectrum of the refugee health burden, but that refugee health data is often conflicting and difficult to interpret and compare and can provide only a ‘snapshot’ of a single moment in the refugee resettlement experience [3]. Longitudinal approaches that collect self-rated general health information from refugees may be one way to overcome this knowledge gap.

Self-rated general health (SRGH) is the subjective measurement of an individual’s general health and is based upon a simple question in which respondents are asked to rate their general health during the past 4 weeks on a scale ranging from “excellent” to “very poor” [10]. Despite its simplicity, SRGH has been found to be an unusually strong predictor of mortality, morbidity and health service utilization [10]. For example, a recent meta-analysis found that those who report “poor” health have a twofold higher risk of all-cause mortality relative to those who report “excellent” health [10]. In ways that are still unclear, SRGH integrates biological, mental, social and functional aspects of a person, including individual and cultural beliefs and health behaviours [11, 12]. Therefore, SRGH can capture information about an individual’s health and well-being that more objective measures cannot. Given that refugees have been shown to rate their health as fair-to-poor more frequently than other immigrants after arrival in a developed country [13], we propose that SRGH may be a valuable health index for use among refugee populations; especially given that SRGH has proven reliability and validity among refugee populations [3].

To address the empirical gap in the refugee health literature, this present study examined the association between self-rated general health and pre- and post-migration experiences of resettled adult humanitarian refugees living in Australia. We used the first three years of data from the Australian Government’s ‘Building A New Life in Australia’ (BNLA) longitudinal survey. Other analyses of the BNLA survey have been published [4, 14–17], but none have investigated the impact of migration on the self-rated health of the BNLA respondents. To our knowledge, this is the first study of its kind to use the described methodological approach to investigate the health of resettled refugees. It is anticipated that the knowledge that emerges from this study will lead to recommendations for resettlement services that might be more effective than those currently in place. The aim of our study is to gain insight into the impact of migration on the long-term general health of refugees during their initial resettlement in Australia.

## Methods

### Data sources

We conducted a secondary examination of the first three waves of the BNLA study, collected from October 2013 to March 2016. The BNLA is a five-year national study (2013–2018) conducted by the Australian Government’s Institute of Family Studies to examine how humanitarian refugees settle into a new life in Australia [18]. The Australian Federal Government’s Department of Social Services (DSS) funded the BNLA study. Further information about the BNLA study design can be found in publicly available documents [18].

### Ethics approval

The BNLA study received ethics approval by the Australian Institute of Family Studies Human Research Ethics Committee. De-identified BNLA data is accessible by authorized researchers who have obtained permission from the Australian Department of Social Services. This permission was obtained by study authors AD, JE and GR. Ethics exemption to use the data was granted by the Monash University Research Ethics Committee (see Additional file 1).

### Study population and sampling

The BNLA cohort comprised of individuals aged 15 years and over who had been granted a permanent humanitarian visa by the Australian Government in the 3–6 months preceding the baseline BNLA study [18]. Eligible participants were identified via the Australian Department of Immigration and Border Protection settlement database from eleven locations around Australia to ensure adequate sample size. Migrating Units (MU) were the primary sampling units for the study and consisted of principal applicants (PA) and secondary applicants (SA). Principal Applicants were the lead participant for the study, and were the initial individuals contacted for participation. Secondary Applicants comprised other members of the Migrating Unit listed on the Principal Applicant’s visa application. The Principal Applicant had to consent to participating before SAs could be invited to do so [18]. A total of 2031 out of 4035 eligible Principal Applicants were contacted and 1509 Principal Applicants and 890 Secondary Applicants completed baseline interviews. The respondents are from 35 different countries and speak 50 different languages and at baseline, aged between 15 and 83 years [18].

### Data collection

The first three waves of ‘Building A New Life in Australia’ data were obtained using either home visits (Waves 1 and 3) or telephone interviews (Wave 2). The BNLA written survey was translated into 14 different languages for Wave 1. A total of 19 languages were covered for the survey with the aid of interpreters [18].

### Variable selection

A number of predictor variables were selected from the BNLA survey and related to migration and resettlement, socio-demographics and the presence/absence of chronic conditions.

**Fig. 1:**
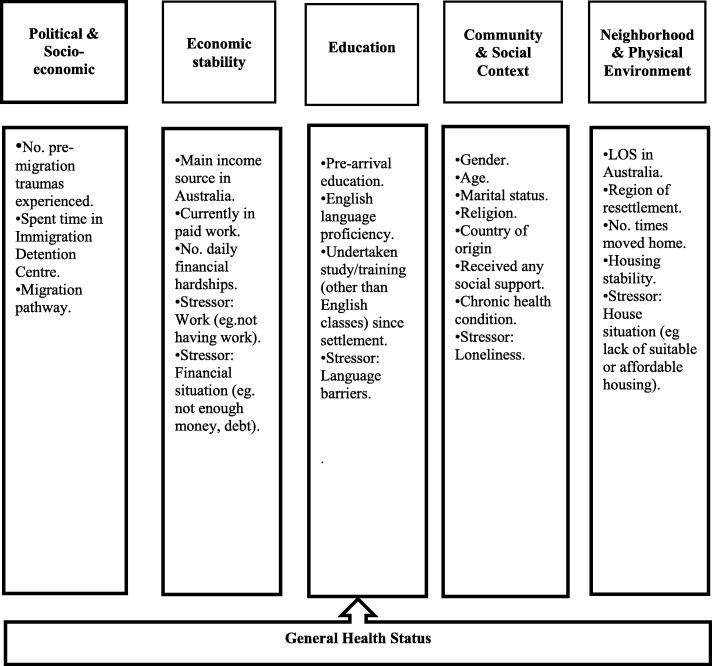
Conceptual framework for the social determinants of health in resettled refugee populations (authors’ own figure)

A conceptual framework used for this study was informed by Cross-Denny & Robinson’s social determinants of health model [19]. The Cross-Denny & Robinson’s model uses five key areas or determinants that are particularly relevant for oppressed and marginalised populations. We added “Political, Socio-Economic” category to encompass the significant associations between structural/political factors (such as spent time in immigration detention centres, migration pathway) and poorer health among refugees. Our adapted model includes six key determinants of refugee health and 25 candidate variables (See Fig. 1). A review of the literature reporting predictors of refugee health outcomes informed the selection of candidate variables from the BNLA dataset to populate the social determinant of health model.

### Outcome measures

Factors relating to migration and resettlement in host country and health were measured by the ‘Building A New Life In Australia’ self-report survey [18]. Our principal outcome variable was self-rated general health and used the question “Overall, how would you rate your health during the past 4 weeks?” selected from the SF-36 [20]. The SF-36 is a generic measure of health status with reliability and validity among refugee populations [3]. The BNLA survey uses a 6-point Likert response scale (*“Excellent”* to *“Very Poor”).* For our study, we use a dichotomous variable comprising of two health response categories of *“Excellent-Good”* and *“Fair-Very Poor”.* The decision regarding the creation of the two response categories was based on a review of the literature, which confirmed that our dichotomous response groupings were appropriate [21].

### Statistical analysis

Analysis included descriptive and generalized linear mixed modelling (GLMM). Descriptive statistics describe the overall characteristics of the sample population at baseline, such as socio-demographics, migration experiences using means and standard deviations (SDs) for continuous variables and frequencies and percentages for categorical variables. All analyses used adjusted weights, provided by the BNLA project. Variables with over 10% missing data at baseline were excluded if we were unable to account for the missing data. Only variables asked across all three waves were included in the analysis. Continuous outcome variables, such as age, were transformed into categorical variables. All analyses were done with Stata/SE 15.1 [22].

To examine the relationships longitudinally between outcomes and predictor variables, GLMM were used. As the BNLA data involved multi-level, clustered sampling, GLMMs accounted for inter-individual random effects between different ‘migrating unit’ clusters (ie. the families) and individual participants, as well as intra-individual fixed effects or the repeated measures within individual participants. GLMMs were appropriate for our analysis as ordinary linear models are unable to handle clustered data. Variable selection for the final models occurred in several steps. Firstly, univariate regressions were used to examine associations between baseline candidate variables and outcome variables, with variables retained at *p* < 0.1. In the second step, collinearity was then examined using Pearson’s correlation coefficient to remove highly correlated variables from the final model. In this study, one candidate variable was selected from two or more correlated variables when r > 0.5. The third step involved examining associations between candidate variables and outcome variables using multivariate GLMM. A variable denoting study time: ‘wave’ was included in these models. Variable at *p* < 0.1 were retained. In the last step, candidate variables were modelled over 1000 bootstrap samples at 95% resampling for the data set. Candidate variables were retained in final models if they were identified as significant (*p* < 0.05) in 1000 bootstraps over 50% of the time.

### Role of the funding source

The BNLA is funded by the Australian Government’s Department of Social Services and were not involved in the preparation of this manuscript or the analyses reported.

## Results

### Demographics

At baseline, 2399 individuals took part in the BNLA study. The mean age was 35.5 years (SD = 13.9) and 54.0% were male. Over half of the participants (61.0%) were married or partnered and nearly half (45%) were born in the Middle East. Almost a third (32.5%) had little to no schooling upon arrival to Australia and 12.1% held university qualifications. Before coming to Australia, 33.6% could speak English *‘very well-well’.* A vast majority (88.9%) of participants had exposure to one or more traumatic experiences. Mostly, participants had been in Australia less than six months (82.1%) and were living in metropolitan cities (90.7%). There was little financial stability with high unemployment (90.7%); high dependency on government income support (88.0%) and at least one financial daily hardship (42.3%). Only 9.3% were in paid employment at baseline. Stress caused by not having work, stable housing and language barriers was reported by 33.0, 28.1 and 56.0% of participants, respectively. Having a ‘chronic’ health condition was reported by 23.0% of participants at baseline. See Table 1.

**Table 1:** Baseline characteristics of humanitarian refugees in the Building A New Life in Australia project, 2013–14 (weighted data)

Characteristic	Description	Response	Total (*n* = 2399)*
Age, mean (years)	Age	–	35.5 (13.9)
Age category (years)	Age category	14–18	218 (9.1%)
		19–25	523 (21.8%)
		26–35	696 (29.0%)
		36–45	475 (19.8%)
		46–55	272 (11.4%)
		56–65	143 (6.0%)
		65+	69 (2.9%)
Gender	Gender	Male	1295 (54.0%)
		Female	1103 (46.0%)
Married or partnered	Married or has partner	Yes	1375 (61.0%)
		No	879 (39.0%)
Region of birth	Major groups based on the Standard Australian Classification of Countries major groups	Middle East	1073 (45.0%)
		Central Asia	482 (20.2%)
		Southern Asia	332 (14.0%)
		South-East Asia	226 (9.5%)
		Africa	273 (11.4%)
Education level, pre-arrival	Highest level of education achieved prior to arrival in Australia	Never attended school	350 (14.8%)
		< 6 years of school	419 (17.7%)
		6–12 years of school	1171 (49.4%)
		Trade or tech qualification	142 (6.0%)
		University degree	87 (12.1%)
English speaking proficiency	Currently understands spoken English	Very well/well	793 (33.6%)
		Not well/not at all	1571 (66.4%)
No. pre-migration trauma experienced	Number of pre-migration traumas experienced	None	248 (11.2%)
		1–2	1286 (57.9%)
		3 or more	689 (31.0%)
Migration pathway	Arrived in Australia via onshore^†^ or offshore migration pathway^††^	Onshore pathway	458 (19.1%)
		Offshore pathway	1940 (80.9%)
Spent time in offshore Immigration Detention Centre(s)	Spent time in offshore Immigration Detention Centre(s) prior to resettlement	Yes	202 (8.6%)
		No	2146 (91.4%)
Duration of stay in Australia	Length of time spent in Australia	Less than 1 year	1968 (82.1%)
		1–2 years	271 (11.3%)
		2–3 years	76 (3.2%)
		More than 3 years	84 (3.5%)
Region of settlement in Australia	Region of settlement in Australia	Metropolitan cities	2174 (90.7%)
		Inner regional Australia	184 (7.7%)
		Outer regional Australia	39 (1.7%)
Currently in paid employment	Currently in paid employment in Australia	Yes	143 (9.3%)
		No	1403 (90.7%)
Main income source in Australia	Main source of income in Australia	Own salary/income	180 (7.7%)
		Government support	2059 (88.0%)
		Other	101 (4.3%)
No. daily financial hardships	Number of daily financial hardships experienced in Australia	None	1307 (57.8%)
		1–2	654 (29.0%)
		3–4	240 (10.7%)
		5–6	59 (2.6%)
Undertaken further study/training in Australia	Undertaken study or job training in Australia, other than English language classes	Yes	410 (17.4%)
		No	1956 (82.6%)
Stress-language barriers	Language barriers as main source of stress in Australia	Yes	1279 (56.0%)
		No	1009 (44.0%)
Stress-work situation	Work situation as main source of stress in Australia (eg unemployment, hours of work, conditions)	Yes	754 (33.0%)
		No	1534 (67.0%)
Stress-housing situation	Work situation as main source of stress in Australia (eg lack of suitable or affordable housing)	Yes	659 (28.1%)
		No	1629 (71.2%)
Stress-loneliness	Loneliness as main source of stress in Australia	Yes	363 (15.9%)
		No	1926 (84.1%)
Received any social support	Received any religious, like ethnic or community support in Australia	Yes	859 (35.8%)
		No	1539 (64.2%)
Has long term disability, injury or health condition	Presence of absence of long-term health condition that has lasted or is likely to last > 12 mths	Yes	859 (35.8%)
		No	1539 (64.2%)

*Data are provided as n (%) or mean (SD)

^†^Onshore pathway is available to those who wish to apply for asylum after arrival in Australia as an unauthorised maritime arrival or holder of valid visa (eg. tourist)

^††^Offshore pathway is available to those who may be eligible for resettlement to Australia, such as those identified by the UNHCR or those eligible for sponsorship to Australia

### GLMM results

Table 2 provides the results of generalised linear mixed effect models, with general health as the outcome variable and pre- and post-migration factors as the predictor variables across the three years of follow-up. Individual characteristics, such as being female, older in age, originating from the Middle East and having a university qualification were significant positive predictors of poorer general health. For example, our results show that over the three years of follow up, females have 2.02 the odds of reporting ‘Fair-Very Poor’ health compared with males. In terms of post-migration stressors, several economic factors were associated with the reporting of ‘Fair-Very Poor’ health among this refugee population. An increasing number of daily financial stressors was significantly associated with higher odds of reporting poorer health. For example, those with 3–6 daily financial hardships were at increased risk for reporting poorer general health than those without any daily financial stressors. “Other” main income source (ie. spouse/partner/parent’s income) was also positively associated with poorer health (OR: 3.25, 95% CI: 0.013).

**Table 2:** Results from generalised linear mixed models using self-rated general health in past 4 weeks with “Excellent-Good” and “Fair-Very Poor” the outcome. OR-odds ratio; p = p-value; CI = confidence interval

Variable	Response	General Health		
		*OR*	*p*	*95% CI*
Gender	Male	–	–	–
	Female	2.02	0.000	(1.51–2.71)
Age Group (years)	18–25	–	–	–
	26–35	1.71	0.032	(1.05–2.78)
	36–45	3.45	0.000	(2.13–5.60)
	46–55	6.91	0.000	(3.83–5.50)
	56–65	9.86	0.000	(5.00–9.41)
	65+	8.32	0.000	(3.31–10.9)
Region of Birth	Middle East	–	–	–
	Africa	0.39	0.000	(0.17–0.56)
	Central Asia	0.39	0.000	(0.26–0.58)
	South-East Asia	0.92	0.802	(0.50–1.70)
	Southern Asia	1.08	0.755	(0.65–1.81)
Pre-arrival education	No schooling	–	–	–
	≤6 years of schooling	1.15	0.545	(0.73–1.82)
	6–12 years of schooling	0.70	0.118	(0.45–1.09)
	Trade or technical qualification	0.70	0.308	(0.36–1.38)
	University degree	0.50	0.021	(0.28–0.90)
Main income source in Australia	Own salary or wage	–	–	–
	Government support	1.50	0.156	(0.86–2.65)
	Other	3.25	0.013	(1.27–8.30)
No. daily financial hardships	0	–	–	–
	1–2	1.75	0.002	(1.23–2.51)
	3–6	3.01	0.001	(1.60–5.70)
Has long term disability, injury or health condition	Yes	–	–	–
	No	0.15	0.000	(0.09–1.04)

Not having a chronic disability/injury or condition was associated with reporting ‘better’ general health (OR: 0.15; 95% CI 0.09–1.04) among this refugee population. That is, those without a chronic health condition have 0.15 the odds of reporting ‘Fair-Very Poor’ health compared with those with a chronic health condition. Another way to interpret the results is that those who do not have a chronic health condition are 85% less likely to report poorer general health. We found no significant association between the health outcomes and duration of stay in Australia, education, accommodation, social support or discrimination.

### Self-rated general health

The mean general health prevalence among humanitarian refugees was calculated in two ways: (1) using BNLA sample data, and (2) using the final model adjusting for model independent variables. Both methods used the provided sample weights. Both methods showed no significant changes in general health over time. See Table 3.

**Table 3:** Mean general health prevalence among humanitarian refugees in the BNLA project calculated two ways using: (1) sample data; and (2) final model adjusting for model independent variables. Both methods used the provided sample weights. (n)^n^ = Number provided in the sample calculation only

General Health		Wave 1		Wave 2		Wave 3	
*Fair-Very Poor*	Weights applied	%(n)^n^	95% CI	%(n)^n^	95% CI	%(n)^n^	95% CI
Mean calculated from sample	Yes	35.7% (805)	33.8–37.7%	36.8% (691)	34.6–39.0%	39.7% (685)	37.4–42.0%
Mean calculated for model variables	Yes	34.6		34.0		33.4	

NOTE: No significant differences between waves as suggested by 95% confidence intervals and this was confirmed using z-tests: Waves1–2 difference z statistic = 0.28, *p* = 0.779; Waves 2–3 difference z-statistic = 0.25; *p* = 0.802; and Waves1–3 difference z-statistic = 0.66; *p* = 0.51

Using BNLA sample data, 35.7% (33.8–37.7%) of participants reported *“Fair-Very Poor”* self-rated health at baseline (see Table 2). At Wave 2, 691 (36.8%) participants’ self-reported *‘Fair-Very Poor’* general health (95% CI 34.6–39.0%) and 685 (39.7%) had *‘Fair-Very Poor’* general health (95% CI 37.4–42.0%) at Wave 3.

Table 3 shows the prevalence estimates of “Fair-Very Poor” self-rated health calculated using the final GLMM model. In Wave 1, the prevalence for *‘Fair-Very Poor’* general health was 34.6%. In Waves 2 and 3 the prevalence was 34.0 and 33.4%, respectively. This shows that the prevalence of *‘Fair-Very Poor’* general health remained high across the 3 years but did not decrease or increase substantially over time. The significance of change in *‘Fair-Very Poor’* general health over time was also examined using the final GLMM model.

Between Waves 1 and 2, there was no significant decrease in the prevalence of “Fair-Very Poor” self-rated health (*p* = 0.779). See Table 3. Waves 1 and 2 saw no significant differences in “Fair-Very Poor” self-rated health (*p* = 0.802). There was also no significant overall decrease between Wave 1 and 3 for “Fair-Very Poor” self-rated health (*p* = 0.512). Therefore, the results indicate that there was no significant change in “Fair-Very Poor” self-rated health over the three years of follow-up. In summary, on a population level, the point prevalence of “Fair-Very Poor” self-rated health remained high, but no overall significant increase (or decrease) in “Fair-Very Poor” self-rated health was seen over time.

## Discussion

This study, to our knowledge, is the first to examine the long-term general health status of resettled adult humanitarian refugees in Australia. In doing so, we provide novel insights into the refugee experience of health as they adjust to conditions in a new society, including our revelation of a sustained and high prevalence of poorer general health among refugees over time. In addition, the factors associated with this poorer general health have not previously been reported in a longitudinal study of Australian humanitarian refugees. Consequently, this knowledge has the potential to build an evidence base for development of improved settlement policies and programs, such as targeted health care and health promotion [3].

The high and sustained prevalence of poorer general health suggests that our refugee population bears a substantial health burden compared with the wider Australian population. For example, by comparison, 4% of the Australian population self-report poor general health and 10% of Australians rate their general health as fair [23]. Factors associated with refugee status are plausible explanations for this observed health disparity. For example, there is evidence to suggest that refugees in Australia experience multilevel barriers to the healthcare and this may be affecting their overall health outcomes [24, 25]. Although Australia provides generous support to humanitarian entrants, our results suggest that existing policies on refugee resettlement have been less effective than desired on improving the health of Australian refugees.

Consistent with other research is our finding of an association between post-settlement economic stressors and poorer general health [13, 26, 27]. We also confirm a strong dose-response like relationship with daily financial hardships and poorer general health, however, this relationship has only been shown previously in cross-sectional studies of refugee populations [26]. Unemployment was not a significant predictor of poorer general health, which suggests that it is the financial consequences of unemployment which may be more important for the long-term general health among our refugee population. Nowhere in the literature has an association between main income and poorer general health been reported previously and our finding that ‘other’ main income source (ie. spouse/partner/parent’s income) is interesting because humanitarian refugees in Australia can access welfare payments and employment [28].

Although the association found between the presence of chronic health conditions and poorer health among our refugee population is not unexpected [27, 29], we are the first to report this association in a longitudinal study of resettling refugees. Not only do we provide evidence of chronic disease prevalence among newly arrived refugees, but we also demonstrate its impact on long-term health. Our finding also confirms the reports of the reports of the rising prevalence of chronic health conditions in refugees’ regions of origin, particularly the Middle East [30]. Resettling refugees are potentially susceptible to the development of chronic disease due to host nation stressors as well as the adoption of a less health western diet and a more sedentary lifestyle and resettlement stressors [31]. A number of refugee studies have reported that resettlement has been found to increase the risk of obesity and non-communicable diseases, including diabetes and hypertension, due to weight gain and resettlement stressors [32–34]. Left unmanaged, the chronic health burden among refugee populations is also likely to impact their resettlement success.

Female gender, older in age, Middle Eastern origin and education level were all found to be risk factor for poorer general health, confirming previous cross-sectional studies of refugee populations [35–37]. Previous research has shown that older refugees are particularly vulnerable to physical deterioration post-settlement [35] and there is solid support that poorer health has a higher prevalence among women [8]. Having a university degree (pre-migration) was significantly and negatively associated with long-term self-rated general health among refugees [35, 37]. Among refugee populations, it has been shown that those with lower levels of education are less likely to seek healthcare, participate in health promoting and disease promoting activities [38]. Therefore, it may be that those with a university qualification possess the skills to achieve a higher level of self-sufficiency with regards to their long-term health. That is, they may know their health needs better and are able to access health services better. Given that many refugees sub-groups, such as female Afghanis, have little to no schooling [39], our result highlights the importance of understanding the education needs of resetting refugees as a way of offsetting adverse health outcomes.

## Conclusion

Our study provides an insight into the long-term general health of resettling refugees in Australia and its associated risk factors. Our results highlight a pattern of additional health needs existing within this refugee population. The risk-factors for poorer self-rated health give shape to future interventions or policy responses to help in the health and well-being of this highly vulnerable group.

### Limitations

There are several methodological limitations in this study. Retrospective reporting and reliance on self-reporting may run a risk of not remembering or misrepresenting the events and non-accurate measurement of symptoms [40], presenting a risk of recall bias, personal interpretations across languages and cultures. Further, the BNLA study collected information that is more comprehensive from principal visa applicants than from secondary applicants, which constrains the depth and breadth of findings of the study. Alternating face-to-face with telephone interviews may have introduced some inconsistencies across waves in that some respondents may have answered some questions differently depending on the interview style. Despite these limitations, this study has contributed to the literature by providing information about the migration experiences of a large and ethnically diverse cohort of refugees during their first three years of resettlement in Australia and the impact of such experiences on general health.

### Implications of the findings

Our results show that there is persisting high prevalence of poorer general health among adult refugees across the first three years of their resettlement in Australia. This finding suggests unmet health needs which may be compounded by the challenges of resettlement in a new society. This result highlights the need for increased clinical awareness of this sustained health burden to help inform and prepare refugee health care and settlement service providers. It also underscores the importance for health professionals to consider broad health issues among refugee populations and highlights the potential value of the Australian Medical Benefit Schedule subsidy of refugee health assessments for recent refugee arrivals [41]. The association between poorer general health and settlement economic stressors highlight the importance of understanding and eliminating the barriers, which lead to the high rates of unemployment among refugees, as a way of preventing negative health consequences. Qualitative research into the barriers to employment, using the perspectives of refugees themselves; would be a valuable addition to the current knowledge gap. This study also highlighted that attention should be given to assessing the specific needs of groups of refugees based on their demographic characteristics; for example, older, Middle Eastern females. Given the vulnerabilities of these groups, sustained targeted interventions upon entry to Australia would be beneficial to offset any further decline in health.

## Additional file

Additional file 1: Monash University Human Research Ethics Committee exemption letter regarding the use of the ‘Building A New Life in Australia’ data sets. (DOCX 27 kb)

## Data Availability

All datasets used in this study are available from the Australian Government’s Department of Social Services at the website: https://www.dss.gov.au/national-centre-for-longitudinal-data-ncld/access-to-dss-longitudinal-datasets.

## Abbreviations

AIHW: Australian Institute of Health and Wellbeing
BNLA: Building A New Life In Australia
CI: confidence interval
DSS: Department of Social Services
GLMM: Generalized linear mixed modelling
MU: Migrating Units
OR: odds ratio
PA: Principal applicants
SA: Secondary applicants
*SF-36*: *36*-Item Short Form Health Survey
SRGH: Self-rated general health
UNHCR: United Nations High Commissioner for Refugees
