# Supplementary material for: The association of migration experiences on the self-rated health status among adult humanitarian refugees to Australia: an analysis of a longitudinal cohort study
Source: Int J Equity Health. 2019 Aug 22;18:130. doi: 10.1186/s12939-019-1033-z (PMC6704614; doi:10.1186/s12939-019-1033-z)
Supplement: Supplementary file 1 — Monash University Human Research Ethics Committee exemption letter regarding the use of the ‘Building A New Life in Australia’ data sets. (DOCX 27 kb) [file 12939_2019_1033_MOESM1_ESM.docx]

**Additional file 1: Monash University Human Research Ethics Committee exemption letter regarding the use of the ‘Building A New Life In Australia’ data sets.**


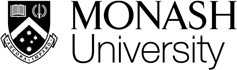


Monash University Human Research Ethics Committee (MUHREC)

26 April 2018

Dear Alison,

**RE: USING THE BUILDING A NEW LIFE IN AUSTRALIA DATA**

Thank you for your email dated 23 April 2018 in regards to using the above data for research.

This is to advise that ethics approval is not required to use the above data as this was collected for use in research and is available by request only.

Please contact me if you require further information.

Sincerely


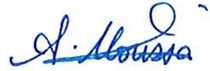


Dr Souheir Houssami

Executive Officer

Human Research Ethics

Cc: Professor Grant Russell, Dr Joanne Enticott

**Human Ethics Office**

Monash University

Room 116, Chancellery Building D

24 Sports Walk, Clayton Campus, Wellington Rd, Clayton VIC 3800, Australia

T: +61 3 9905 2052 Facsimile +61 3 9905 3831 E: muhrec@monash.edu

http://www.intranet.monash/researchadmin/start/ethics/human

ABN 12 377 614 012 CRICOS Provider 00008C
